# The apolipoprotein E polymorphism and the cholesterol-raising effect of coffee

**DOI:** 10.1186/1476-511X-3-26

**Published:** 2004-11-30

**Authors:** Elisabeth Strandhagen, Henrik Zetterberg, Nibia Aires, Mona Palmér, Lars Rymo, Kaj Blennow, Dag S Thelle

**Affiliations:** 1 the Cardiovascular Institute, Department of Medicine, Sahlgrenska University Hospital/Östra, Göteborg, Sweden; 2 Department of Clinical Chemistry and Transfusion Medicine, Sahlgrenska University Hospital/Sahlgrenska, Göteborg, Sweden; 3 Institute of Clinical Neuroscience, Department of Experimental Neuroscience, Sahlgrenska University Hospital/Mölndal, Mölndal, Sweden

**Keywords:** controlled study, *APOE *polymorphism, serum lipids, filtered coffee

## Abstract

**Background:**

The response of serum cholesterol to diet may be affected by the apolipoprotein E (*APOE*) ε2/ε3/ε4 polymorphism, which also is a significant predictor of variation in the risk of coronary heart disease (CHD) and CHD death. Here, we test the hypothesis that the *APOE *polymorphism may modulate the cholesterol-raising effect of coffee.

**Objective:**

We determined the effect of a coffee abstention period and a daily intake of 600 mL coffee on serum cholesterol and triglycerides with respect to the *APOE *polymorphism.

**Design:**

121 healthy, non-smoking men (22%) and women (78%) aged 29–65 years, took part in a study with four intervention periods: 1 and 3) a coffee free period of three weeks, 2 and 4) 600 mL coffee/day for four weeks.

**Results:**

*APOE ε*2 positive individuals had significantly lower total cholesterol concentration at baseline (4.68 mmol/L and 5.28 mmol/L, respectively, p = 0.01), but the cholesterol-raising effect of coffee was not influenced significantly by *APOE *allele carrier status.

**Conclusions:**

The *APOE ε *2 allele is associated with lower serum cholesterol concentration. However, the *APOE *polymorphism does not seem to influence the cholesterol-raising effect of coffee.

## Introduction

Apolipoprotein E (apoE) is a structural component of triglyceride-rich lipoproteins, chylomicrons, very-low-density lipoproteins (VLDL), and high-density-lipoproteins (HDL) [1]. Variation in the *APOE *gene sequence results in the 3 common alleles (*ε2, ε3 *and *ε4*), which can produce 6 different genotypes (ε2/ε2, ε2/ε3, ε2/ε4, ε3/ε3, ε3/ε4 and ε4/ε4). The *ε2, ε3 *and *ε4 *alleles encode three distinct forms of apoE (E2, E3 and E4) and have approximate frequencies of 8%, 77%, and 15%, respectively, in white populations [2]. ApoE3 seems to be the normal isoform in all known functions, while apoE4 and apoE2 can each be dysfunctional [3,4]. In most populations, individuals with the *APOE ε2 *allele display lower levels of plasma cholesterol compared with individuals carrying the *APOE ε3 *allele, whereas individuals with the *APOE ε4 *allele show higher levels of plasma cholesterol, especially LDL-cholesterol [1,2,5]. Subjects with *APOE *ε3/ε4 and ε4/ε4 genotypes absorb cholesterol effectively and have higher non-fasting serum triglyceride values than ε4 negative individuals [6,7]. The allelic variation in the *APOE *gene is shown to be a significant predictor of variation in the risk of coronary heart disease (CHD) and CHD death [2-4,8-10], but the results from an extensive prospective study showed no associations [11]. Both the MONICA Project [12] and the Scandinavian Simvastatin Survival Study [13] suggest an increased risk of CHD for individuals carrying the *APOE *ε*4 *allele. The *APOE *ε*4 *allele is also considered a strong risk factor for Alzheimer's disease [14-16].

The serum cholesterol-raising effect of coffee is due to the diterpenes kahweol and cafestol [17]. Earlier studies have shown a cholesterol-raising effect mainly of unfiltered coffee, because a major part of the diterpenes is retained by a paper filter [18-20]. A recent trial, however, indicates that filtered coffee has a more pronounced serum cholesterol-raising effect than previously anticipated [21]. This finding was further corroborated in a randomized intervention study, where we demonstrated a considerable cholesterol-raising effect of filtered coffee [22]. In the study two coffee abstention periods were associated with a significant decline in serum cholesterol of 0.22 and 0.36 mmol/L, respectively, while 600 mL filtered coffee a day during two different periods increased serum cholesterol by 0.25 and 0.15 mmol/L, respectively. Here, we test the hypothesis that these effects might be modulated by the *APOE ε2*/*ε3*/*e4 *polymorphism.

## Subjects and methods

### Trial design

The study was organised as a prospective, controlled study with four consecutive trial periods. The first and third periods were 3 weeks of total coffee abstention. The second and fourth period consisted of 4 weeks with the subjects consuming 600 mL filter brewed coffee/day.

**Figure 1:**
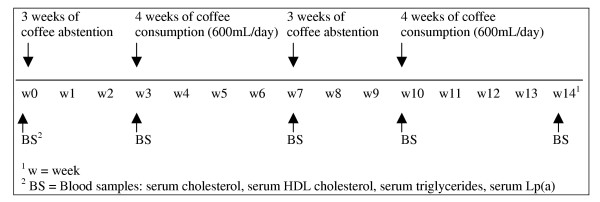
Study design

The main outcome or effect variable was total serum cholesterol and the effect was assessed as the difference between the measurements at the beginning and the end of the coffee free periods (coffee abstention) and the difference between measurements at the beginning and at the end of the four weeks of coffee consumption (Figure 1). Trial duration of 3–4 weeks has previously been shown to be sufficient to get an effect of coffee on serum cholesterol [21,23].

### Subjects and procedure

Participants were recruited by advertising in Gothenburg's major newspaper. Inclusion criteria were age-range 30–65 years, free from clinically recognized chronic diseases, such as cardiovascular diseases, cancer, renal disorders, liver disease and diabetes mellitus. The participants were required to be free from anti-epileptic or cholesterol lowering drugs, and had been using coffee on a regular basis for at least five years and were currently non-smokers (at least for the last six months).

During the coffee drinking periods the participants were instructed to drink about 600 mL filter brewed coffee/day (4 cups), according to standardised measures. The coffee was provided to guarantee that they were all exposed to the same brand and quality of filter brewed coffee. All participants also got the same kind of standardised coffee filter and measuring spoon.

The coffee filters used were of the brand Euro-Shopper, made by Indupa N.V., Zaventem, Belgium. Divergence from the 4 cups was reported. The participants were allowed to drink tea and other caffeine containing beverages during the coffee-free periods.

### Effect variables

Non-fasting blood samples were drawn at inclusion and at three, seven, ten and fourteen weeks after start of the study. Prior to analysis, prepared serum was stored at -70°C.

The blood samples were analysed for blood lipids (total cholesterol, HDL cholesterol, triglycerides, lipoprotein(a) (Lp(a)) and urate in serum. Serum cholesterol and triglycerides were determined by an enzymatic procedure on a Hitachi 917 analyzer. HDL cholesterol was determined enzymatically after precipitation of VLDL, LDL and chylomicrones by α-cyklodextrinsulphate and dextransulphate. Determination of Lp(a) was done by the method Tint Elize Lp(a) of Biopool International. Serum urate was analysed by Hitachi 917 autoanalyser. Body Mass Index (BMI; kg/m^2^) was recorded once during the study. Blood pressure was recorded by manual device and EKG and heart rate were recorded at all five visits.

The dietary habits were assessed by dietary frequency questionnaires at the beginning of the study. A follow-up survey with special emphasis on changes in food habits during the four different periods was undertaken. The dietary questionnaire was based upon a Norwegian version, which has been used in a number of previous studies [24].

### Genotype Analysis

*APOE *genotypes were determined by solid-phase minisequencing as previously described by Blennow et al [25].

### Statistical methods

All analyses were performed using the SAS^©^software version 8. Signed rank test was used to test differences in the groups. Wilcoxon rank sum test was used to test differences at baseline and differences between the groups. The mean was used as location measure and measures of variation were described in terms of standard deviation. P-values < 0.05 were considered statistically significant.

## Results

A total of 156 persons responded to the advertisement and of these 124 fulfilled the criteria and were able to take part. Three persons decided to withdraw during the study, leaving a total of 121 participants. One person was not able to take part during the first two periods and five persons were not able to take part in the last two periods, which resulted in 120 participants in the first trial period and 116 in the subsequent trial period.

### Genotype frequencies

The *APOE *allele frequencies were 6.1% for the ε2 allele, 75.6% for the ε3 allele and 18.2% for the ε4 allele. This distribution agrees well with those reported in other populations in northern Europe [2,3]. Genotype and allele frequencies for the *APOE *polymorphism are given in Table 1.

**Table 1:** *APOE *genotype and allele frequency, n = 121

	n	%
*ε2/ε2*	2	1.7
*ε2/ε3*	9	7.4
*ε2/ε4*	2	1.7
*ε3/ε3*	69	57.0
*ε3/ε4*	36	29.8
*ε4/ε4*	3	2.5
*ε2*	15	6.2
*ε3*	183	75.6
*ε4*	44	18.2

### Serum cholesterol concentrations according to genotype and coffee exposure

Individual *APOE *genotypes (six subgroups, Table 1) did not influence baseline values or coffee-induced changes in serum cholesterol, serum HDL cholesterol, serum triglycerides or serum Lp(a), possibly due to a small sample size (data not shown). ε4-positive individuals had similar serum cholesterol levels and coffee-induced changes in cholesterol concentration as ε4-negative individuals (data not shown). However, when grouping *ε2*-positive individuals it was revealed that these displayed significantly lower cholesterol at baseline (Table 2). There was a significant difference in cholesterol decrease between week 0 and 3 for both groups. There was no difference between the two groups regarding the cholesterol decrease in the first coffee abstention period but there was a significant difference in cholesterol decrease in the second coffee abstention period, where ε*2*-negative individuals displayed a larger decrease in cholesterol (Table 2).

**Table 2:** Serum cholesterol concentration (mmol/L) at baseline and after two 3-week periods of coffee abstention (week 0 – 3 and week 7 – 10) for *APOE *ε*2*-positive (n = 13) and *APOE *ε*2*-negative (n = 108) individuals

	***APOE ε2*-positive**	***APOE *ε*2*-negative**	**p**
	n = 13	n = 107/103 ^a^	
*First trial period*			
week 0	4.68 (0.80)	5.28 (0.93)	0.01^b^
week 3	4.49 (0.71)	5.05 (0.90)	
diff week 0–3	-0.18 (0.24)	-0.23 (0.55)	0.30 ^c^
p (diff 0–3)	0.02 ^d^	<0.0001^d^	
*Second trial period*			
week 7	4.52 (0.71)	5.34 (0.93)	
week 10	4.34 (0.64)	4.95 (0.89)	
week 7–10	-0.18 (0.41)	-0.39 (0.55)	0.08 ^c^
p (diff 7–10)	0.13	<0.0001^d^	

^a ^107 participants in the first trial period and 103 participants in the second trial period

^b ^Significant difference between *APOE *ε*2*-positive and *APOE *ε*2*-negative at baseline, Wilcoxon rank sum test

^c ^No significant difference between *APOE *ε*2*-positive and *APOE *ε*2*-negative in differences between week 0–3 or week 7–10, Wilcoxon rank sum test

^d ^Significant difference between week 0–3 for the *APOE *ε*2*-positive group and between week 0–3 and week 7–10 for the *APOE *ε*2*-negative group, Signed rank test

Coffee consumption resulted in a significant cholesterol increase in the ε*2*-negative group in both trial periods (w 3–7 and w 10–14), but not in the ε*2*-positive group (Table 3). There were no differences between the ε*2*-positive and the ε*2*-negative group according to baseline characteristics as sex, age, body mass index (BMI) and coffee consumption prior to the study (Table 4).

**Table 3:** Serum cholesterol concentration (mmol/L) after two 4-week periods of coffee consumption (week 3 – 7 and week 10 – 14) for *APOE *ε*2*-positive (n = 13) and *APOE *ε*2*-negative (n = 108) individuals

	*APOE *ε*2*-positive	*APOE *ε*2*-negative	p
	n = 13	n = 107/103 ^a^	
*First trial period*			
week 3	4.49 (0.71)	5.05 (0.90)	
week 7	4.52 (0.71)	5.34 (0.93)	
diff week 3–7	0.03 (0.57)	0.29 (0.57)	0.09 ^b^
p (diff 3–7)	0.70	<0.0001 ^c^	
*Second trial period*			
week 10	4.34 (0.64)	4.95 (0.89)	
week 14	4.54 (0.84)	5.09 (0.85)	
diff week 10–14	0.20 (0.47)	0.14 (0.59)	0.37 ^b^
p (diff 10–14)	0.15	0.009 ^c^	

^a ^107 participants in the first trial period and 103 participants in the second trial period

^b ^No significant difference between 2-positive and 2-negative in differences between week 3–7 or week 10–14, Wilcoxon rank sum test

^c ^Significant difference between week 0–3 and week 7–10 for the *APOE *ε*2*-negative group, Signed rank test

**Table 4:** Baseline characteristics for *APOE *ε*2*-positive (n = 13) and *APOE *ε*2*-negative (n = 108) individuals

	*APOE *ε*2*-positive	*APOE *ε*2*-negative	p
	n = 13	n = 108	
Sex (% women)	77	79	ns ^a^
Age (years)	46.6	48.7	0.44 ^b^
BMI (kg/m^2^)	25.7	25.8	0.87 ^b^
Coffee consumption (cups/day)	4.3	3.7	0.12 ^b^

^a ^No significant difference between *APOE *ε*2*-positive and *APOE *ε*2*-negative at baseline, Chi square test

^b ^No significant difference between *APOE *ε*2*-positive and *APOE *ε*2*-negative at baseline, Wilcoxon rank sum test

### Dietary monitoring and compliance

The dietary survey did not reveal any important changes during the four intervention periods [22]. Coffee consumption or non-compliance was reported by six persons during the first coffee abstention period (mean 1.8 cups/period), whereas four persons reported coffee consumption in the second coffee abstention period (mean 0.7 cups/period).

## Discussion

Subjects with different *APOE *genotypes differ in the absorption efficiency of cholesterol from the intestine, in the synthesis rates of cholesterol and bile acids, and in the production of LDL apoprotein B [3,26]. This suggests that the response of serum cholesterol to diet may be affected by the *APOE e2/e3/e4 *polymorphism [27,28]. One previous study examined the effect of purified cafestol on serum lipids in relation to the *APOE *polymorphism [26] and found that responses of LDL-cholesterol to cafestol were slightly smaller among carriers of the *APOE *ε4 allele. Here, we investigate for the first time the possible influence of the *APOE *polymorphism on the cholesterol-raising effect of filtered coffee.

*APOE *ε4-positive individuals did not differ significantly from ε4-negative individuals with regard to baseline cholesterol concentration or coffee-induced changes in cholesterol concentration. However, we confirm that ε2-positive individuals display significantly lower cholesterol levels at baseline than ε2-negative individuals. A tendency was seen that *ε2*-positive individuals might be partly protected from the cholesterol increasing effect of coffee consumption. This was, however, only seen in the first trial period and will require further investigations. In conclusion, the *APOE ε2*/*ε3*/*ε4 *polymorphism is not a strong modulator of the cholesterol-increasing effect of coffee. Other genes should be discussed and further investigation is needed to see if there is a genetic factor in the cholesterol-raising effect of coffee.

## References

[B1] Ou T, Yamakawa-Kobayashi K, Arinami T, Amemiya H, Fujiwara H, Kawata K, Saito M, Kikuchi S, Noguchi Y, Sugishita Y, Hamaguchi H (1998). Methylenetetrahydrofolate reductase and apolipoprotein E polymorphisms are independent risk factors for coronary heart disease in Japanese: a case-control study. Atherosclerosis.

[B2] Davignon J, Gregg RE, Sing CF (1988). Apolipoprotein E polymorphism and atherosclerosis. Arteriosclerosis.

[B3] Eichner JE, Dunn ST, Perveen G, Thompson DM, Stewart KE, Stroehla BC (2002). Apolipoprotein E polymorphism and cardiovascular disease: a HuGE review. Am J Epidemiol.

[B4] Mahley RW, Rall SC (2000). APOLIPOPROTEIN E: Far More Than a Lipid Transport Protein. Annu Rev Genomics Hum Genet.

[B5] Song Y, Stampfer MJ, Liu S (2004). Meta-analysis: apolipoprotein E genotypes and risk for coronary heart disease. Ann Intern Med.

[B6] Tammi A, Rönnemaa T, Rask-Nissilä L, Miettinen TA, Gylling H, Valsta L, Viikari J, Välimäki I, Simell O (2001). Apolipoprotein E Phenotype Regulates Cholesterol Absorption in Healthy 13-Month-Old Children-The STRIP Study. Pediatr Res.

[B7] Tammi A, Rönnemaa T, Viikari J, Jokinen E, Lapinleimu H, Ehnholm C, Simell O (2000). Apolipoprotein E4 phenotype increases non-fasting serum triglyceride concentration in infants – the STRIP study. Atherosclerosis.

[B8] Stengård JH, Pekkanen J, Ehnholm C, Nissinen A, Sing CF (1996). Genotypes with the apolipoprotein ε4 allele are predictors of coronary heart disease mortality in a longitudinal study of elderly Finnish men. Hum Genet.

[B9] Ilveskoski E, Perola M, Lehtimäki T, Laippala P, Savolainen V, Pajarinen J, Penttilä A, Lalu KH, Männikkö A, Liesto KK, Koivula T, Karhunen PJ (1999). Age-dependent association of apolipoprotein E genotype with coronary and aortic atherosclerosis in middle-aged men. An autopsy study. Circulation.

[B10] Corbo RM, Scacchi R, Vilardo T, Ruggeri M (2001). Polymorphisms in the Apolipoprotein E gene regulatory region in relation to coronary heart disease and their effect on plasma Apolipoprotein E. Clin Chem Lab Med.

[B11] Liu S, Ma J, Ridker PM, Breslow JL, Stampfer MJ (2003). A prospective study of the association between APOE genotype and the risk of myocardial infarction among apparently healthy men. Atherosclerosis.

[B12] Stengård JH, Weiss KM, Sing CF (1998). An ecological study of association between coronary heart disease mortality rates in men and the relative frequencies of common allelic variations in the gene coding for apolipoprotein E. Hum Genet.

[B13] Gerdes LU, Gerdes C, Kervinen K, Savolainen M, Klausen IC, Hansen PS, Kesaniemi YA, Faergeman O (2000). The apolipoprotein  allele determines prognosis and the effect on prognosis of simvastatin in survivors of myocardial infarction. A substudy of the Scandinavian Simvastatin Survival Study. Circulation.

[B14] Corder EH, Saunders AM, Strittmatter WJ, Schmechel DE, Gaskell PC, Small GW, Roses AD, Haines JL, Pericak-Vance MA (1993). Gene dose of apolipoprotein E type 4 allele and the risk of Alzheimer's disease in late onset families. Science.

[B15] Poirier J, Davignon J, Bouthillier D, Kogan S, Bertrand P, Gauthier S (1993). Apolipoprotein E polymorphism and Alzheimer's disease. Lancet.

[B16] Strittmatter WJ, Saunders AM, Schmechel D, Pericak-Vance M, Enghild J, Salvesen GS, Roses AD (1993). Apolipoprotein E: high-avidity binding to β-amyloid and increased frequency of type 4 allele in late-onset familial Alzheimer's disease. Proc Natl Acad Sci USA.

[B17] Urgert R, Katan B (1997). The cholesterol-raising factor from coffee beans. Annu Rev Nutr.

[B18] Bak A (1990). Coffee and cardiovascular risk; an epidemiological study. PhD thesis.

[B19] Ahola I, Jauhiainen M, Aro A (1991). The hypercholesterolaemic factor of boiled coffee is retained by a paper filter. J Inter Med.

[B20] Van Dusseldorp M, Katan MB, Van Vliet T, Demacker PNM, Stalenhoef A (1991). Cholesterol-raising factor from boiled coffee does not pass a paper filter. Arterioscler Thromb.

[B21] Christensen B, Mosdøl A, Retterstøl L, Landaas S, Thelle DS (2001). Abstention from filtered coffee reduces the levels of homocysteine and cholesterol – a randomized, controlled trial. Am J Clin Nutr.

[B22] Strandhagen E, Thelle DS (2003). Filtered coffee raises serum cholesterol: results from a controlled study. Eur J Clin Nutr.

[B23] Grubben MJ, Boers GH, Blom HJ, Broekhuizen R, de Jong R, van Rijt L, de Ruijter E, Swinkels DW, Nagengast FM, Katan MB (2000). Unfiltered coffee increases plasma homocysteine concentrations in healthy volunteers: a randomized trial. Am J Clin Nutr.

[B24] Nes M, Frost Andersen L, Solvoll K, Sandstad B, Hustvedt BE, Løvø A, Drevon C (1992). Accuracy of a quantitative food frequency questionnaire applied in elderly Norwegian women. Eur J Clin Nutr.

[B25] Blennow K, Ricksten A, Prince JA, Brookes AJ, Emahazion T, Wasslavik C, Bogdanovic N, Andreasen N, Batsman S, Marcusson J, Nagga K, Wallin A, Regland B, Olofsson H, Hesse C, Davidsson P, Minthon L, Jansson A, Palmqvist L, Rymo L (2000). No association between the alpha2-macroglobulin (A2M) deletion and Alzheimer's disease, and no change in A2M mRNA, protein, or protein expression. J Neural Transm.

[B26] Weggemans RM, Zock PL, Ordovas JM, Pedro-Botet J, Katan MB (2001). Apoprotein E genotype and the response of serum cholesterol to dietary fat, cholesterol and cafestol. Atherosclerosis.

[B27] Masson LF, McNeill G, Avenell A (2003). Genetic variation and the lipid response to dietary intervention: a systematic review. Am J Clin Nutr.

[B28] Ordovas JM (2002). Gene-diet interaction and plasma lipid responses to dietary intervention. Biochem Soc Trans.

